# Supplementary material for: Effectiveness of Volatile Natural Deep Eutectic Solvents (VNADESs) for the Green Extraction of Chelidonium majus Isoquinoline Alkaloids
Source: Molecules. 2022 Apr 28;27(9):2815. doi: 10.3390/molecules27092815 (PMC9101032; doi:10.3390/molecules27092815)
Supplement: Supplementary file 1 [file molecules-27-02815-s001.zip › molecules-1700242-supplementary.pdf]

molecules

MDPI

Supplementary Materials

Effectiveness of volatile natural deep-eutectic solvents (VNADES) for the green extraction of *Chelidonium majus* isoquinoline alkaloids

Maciej Strzemski[1,*], Sławomir Dresler[1,2], Beata Podkościelna[3], Kamil Skic[4], Ireneusz Sowa[1], Daniel Załuski[5], Rob Verpoorte[6], Sylwia Zielińska[7], Paweł Krawczyk[8], and Magdalena Wójciak [1,*]

[1] Department of Analytical Chemistry, Medical University of Lublin, 4A Chodźki Str., 20-093 Lublin, Poland; slawomir.dresler@umlub.pl (S.D.); i.sowa@umlub.pl (I.S.)
[2] Department of Plant Physiology and Biophysics, Institute of Biological Science, Maria Curie-Skłodowska University, 19 Akademicka Str. 20-033 Lublin, Poland
[3] Faculty of Chemistry, Institute of Chemistry, Maria Curie-Skłodowska University, 2 Marii Curie-Skłodowskiej Str., 20-031 Lublin, Poland; beata-pod@poczta.umcs.lublin.pl (B.P.)
[4] Institute of Agrophysics, Polish Academy of Sciences, 4 Doświadczalna Str., 20-290 Lublin, Poland; k.skic@ipan.lublin.pl (K.S.)
[5] Department of Pharmaceutical Botany and Pharmacognosy, Ludwik Rydygier Collegium Medicum, Nicolaus Copernicus University, 9 Marie Curie-Skłodowska Street, 85-094, Bydgoszcz, Poland; daniel.zaluski@cm.umk.pl (D.Z.)
[6] Natural Products Laboratory, Institute of Biology, Leiden University, 2300RA Leiden, the Netherlands; verpoort@chem.leidenuniv.nl (R.V.)
[7] Department of Pharmaceutical Biology and Biotechnology, Division of Pharmaceutical Biotechnology, Wroclaw Medical University, 50-556 Wroclaw, Poland; sylwia.zielinska@umed.wroc.pl (S.Z.)
[8] Immunology and Genetics Laboratory, Department of Pneumonology, Oncology and Allergology, Medical University of Lublin, 20-093 Lublin, Poland, krapa@poczta.onet.pl (P.K.)
* Correspondence: maciej.strzemski@poczta.onet.pl (M.S.); magdalenawojciak@umlub.pl (M.W.)

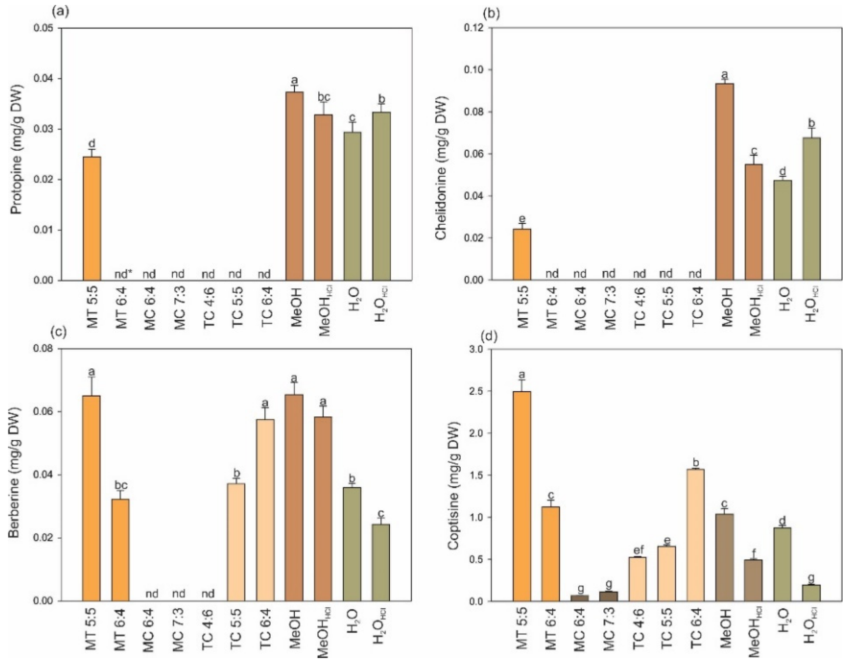

Figure S1. Yields of extraction of isoquinoline alkaloids from *Chelidonium majus* shoots with volatile natural deep-eutectic solvents and commonly used extractants. MT – menthol-thymol mixtures; MC – menthol-camphor mixtures; TC – thymol-camphor mixtures. The mass:mass ratio of the components in the mixtures is shown next to the symbols. $MeOH_{HCl}$ and H_2O_{HCl} – MeOH and water acidified with hydrochloric acid to a concentration of 0.05 M. Data are mean ± SE (n = 5); values for individual raw materials followed by the same letter are not significantly different ($p < 0.05$, Tukey's test).

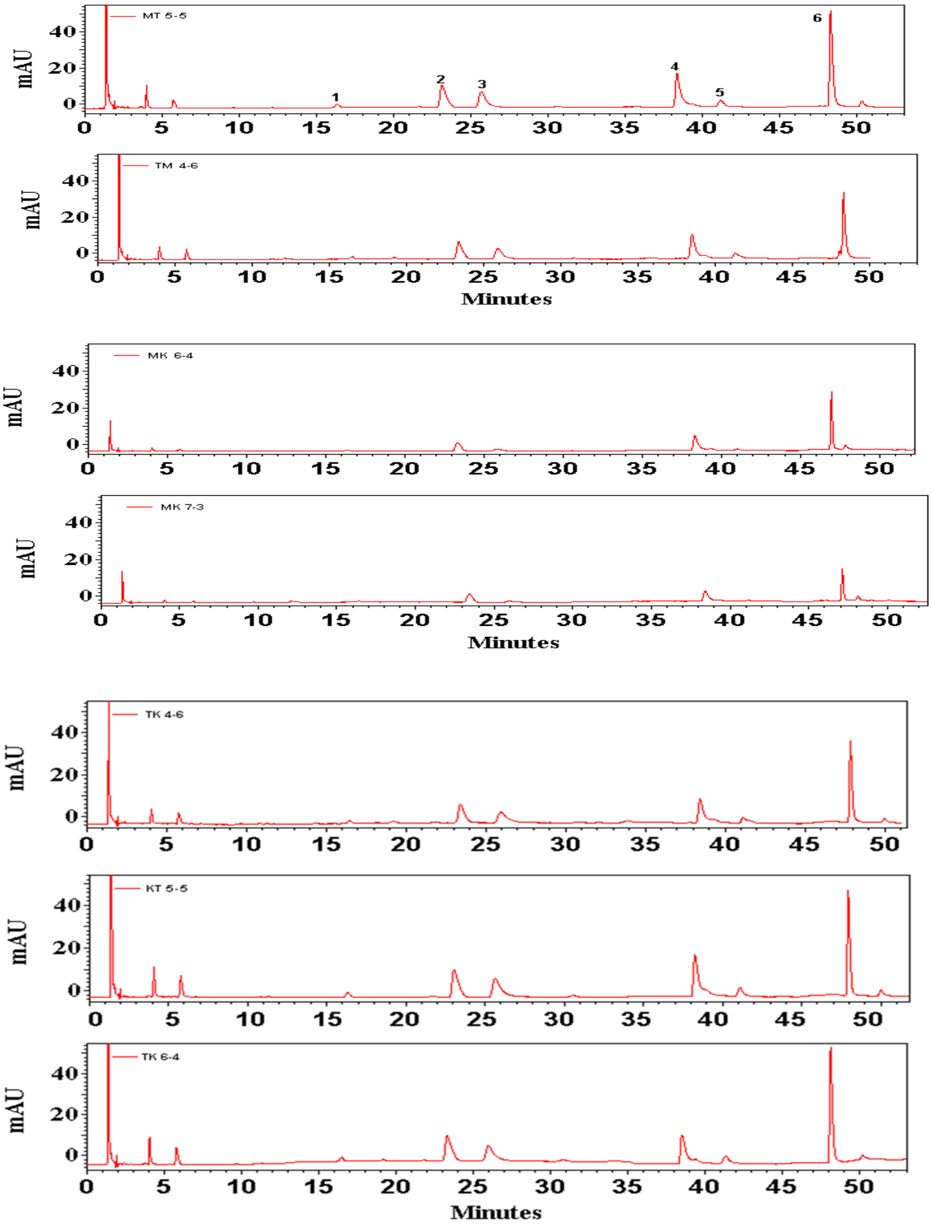

Figure S2. Examples of HPLC-DAD chromatograms of extracts obtained with VNADES (λ=254 nm); 1- protopine, 2- chelidonine 3- coptisine 4- sanquinarine 5- berberine 6- chelerytrine. IA were determined using the methodology published previously [1]: VWR Hitachi Chromaster 600 chromatograph with a spectrophotometric detector (DAD) and EZChrom Elite software (Merck) was used for chromatographic analysisThe samples were analyzed on XB-C18 reversed-phase core-shell column (Kinetex, Phenomenex, Aschaffenburg, Germany) (25 cm × 4.6 mm i.d., 5 µm particle size) using mixture of: acetonitrile (solvent A) and 10mM water solution of ammonium acetate adjusted to pH 4 with acetic acid (solvent B) as mobile phase, at temperature of 30 °C and eluent flow rate 1.2 mL·min^{-1} according to gradient program: A 20%, B 80% during 0-20 min, A 25%, B 75 % during 20-27 min and next A 30%, B 70% during 27- 60 min.

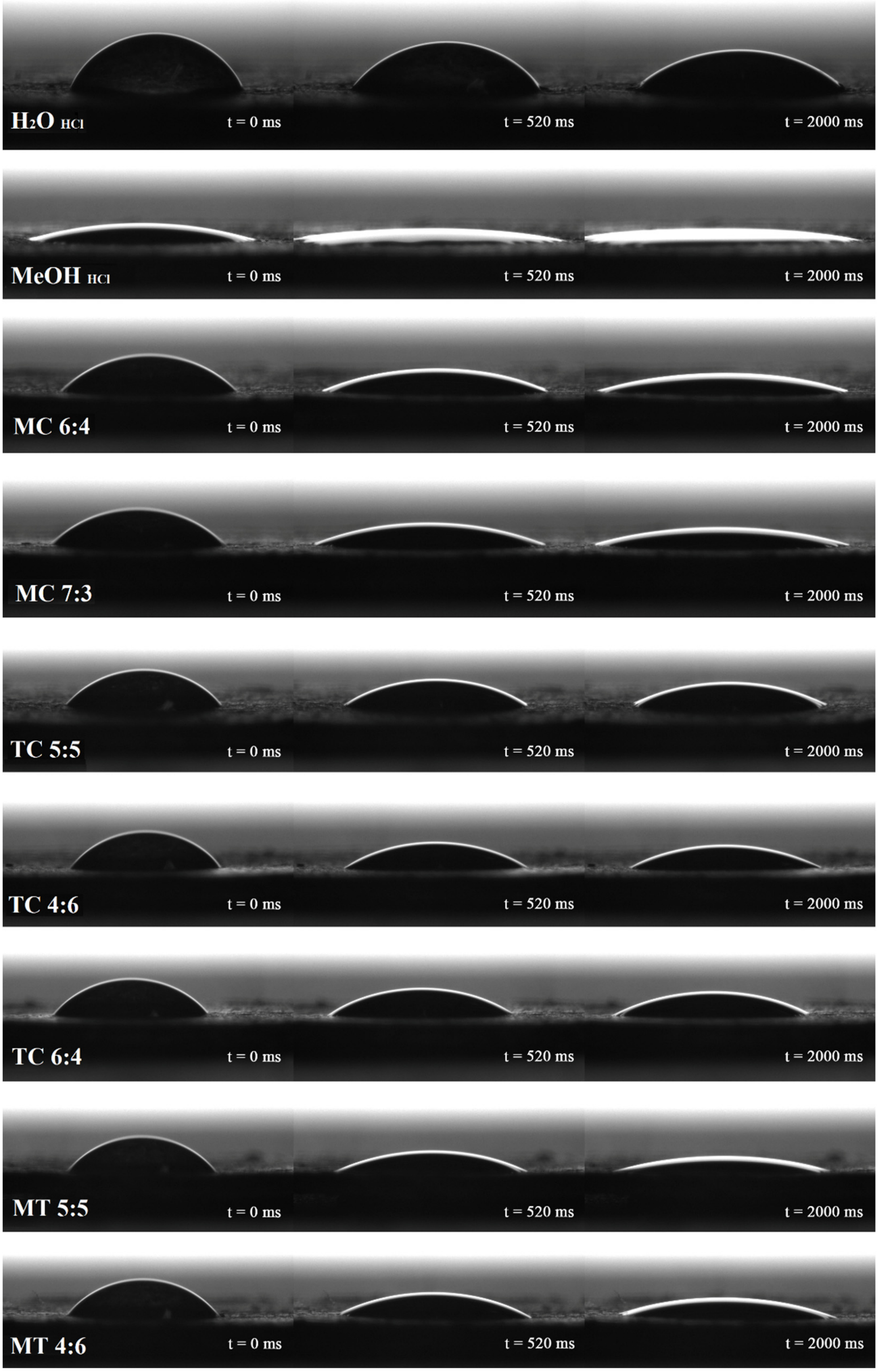

Figure S3. The photographs of drops of applied extractants on the surface of the root *Chelidonium majus*.

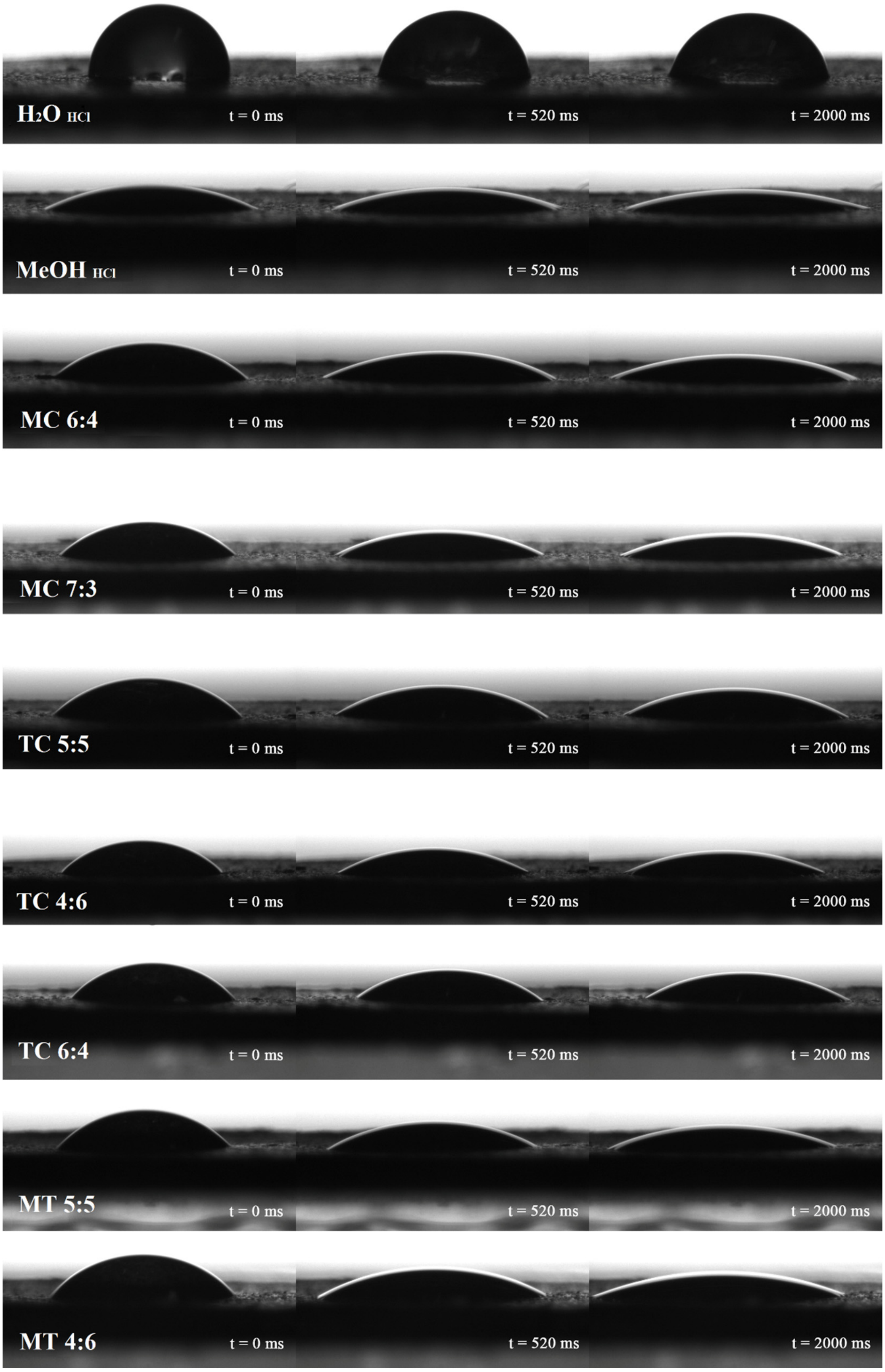

Figure S4. The photographs of drops of applied extractants on the surface of the herb *Chelidonium majus*.

References

1. Wójciak-Kosior, M.; Sowa, I.; Dresler, S.; Kováčik, J.; Staniak, M.; Sawicki, J.; Zielińska, S.; Świeboda, R.; Strzemski, M.; Kocjan, R. Polyaniline based material as a new SPE sorbent for pre-treatment of *Chelidonium majus* extracts before chromatographic analysis of alkaloids. Talanta **2019**, 194, 32–37, doi:10.1016/j.talanta.2018.10.009.
